# Society Meetings

**Published:** 1911-03

**Authors:** 


### SOCIETY MEETINGS

---

THE Medical Society of the State of New York will hold its one hundred and fifth annual meeting Tuesday and Wednesday, April 18 and 19, 1911, under the presidency of Dr. Charles Stover, of Amsterdam. Dr. J. W. Grosvenor, of Buffalo, is first vice-president, and Dr. Wisner R. Townsend, of New York, is secretary.

---

A MEETING of the Committee on Experimental Medicine of the State of New York was held at the Academy of Medicine, New York, on February 7, 1911.

---

THE Rochester Academy of Medicine has elected the following-named officers for 1911:

President, Richard M. Moore; vice-presidents, John R. Williams, E. Wood Ruggles, Arthur W. Thomas, Frank P. Leadley; secretary, William M. Brown, 666 East Avenue; treasurer, Wesley T. Mulligan.

---

THE Homeopathic Medical Society of the State of New York, at its annual meeting, held at Albany, February 14, elected the following named officers for the ensuing year:

President, Orlando S. Ritch, Brooklyn; vice-presidents. George R. Critchlow, Buffalo; Marcena S. Ricker, Rochester;

George H. Jenkins, Binghamton; secretary, Bert B. Clark, New York; treasurer, Reeve B. Howland, Elmira; necrologist, John L Moffatt, Buffalo.

---

The Rochester Academy of Medicine held meetings during January and February, as follows:

*Section II. Obstetrics*—Wednesday evening, January 25. Program: Uterine retrodisplacements, Charles R. Barber. Barber.

*Section I. General Medicine.*—Wednesday evening, February 1. Program: Acute deliriums in psychiatric practice, with special reference to so-called acute delirious mania, E. I Hanes.

*Section II. Surgery.*—Wednesday evening, February 8, 1911. Program: A plea for the early removal of lumps, with report of two cases; Report of a case of inoperable sarcoma treated with Cooley's serum.

*Section III. Obstetrics, Gynecology, and Pediatrics.*—Wednesday evening, February 15. Program: The requirements of modern obstetrics, Franklin S. Newell, Assistant Professor of Obstetrics, Harvard University Medical School.

*Section IV. Public Health.*—Wednesday evening, February 22. Program: Dietary fads—their development and significance (illustrated), J. R. Williams.

---

The Buffalo Academy of Medicine held meetings during January and February as follows:

*Section of Obstetrics and Gynecology.*—Tuesday evening, January 24. Program: A study of children with reference to enteroptosis, illustrated with lantern slides and x-ray plates. Richard R. Smith, Grand Rapids, Mich.

*Section of Pathology.*—Thursday evening, January 26. Program: Tuberculosis, A. H. Caulfield, Gravenhurst, Ont.

*Section of Surgery.*—Tuesday evening, February 7. Program: Some problems in gastrointestinal surgery. J. M. T. Finney, Professor of Surgery, Johns Hopkins University, Baltimore.

*Section of Medicine.*—Tuesday evening, February 14. Program: Claude Bernard, Physiologist, an appreciation, Fred C. Bush; cardiac and pulmonary clinical manifestations of vagus disease, Charles F. Hoover, Cleveland.

*Section of Pathology.*—Tuesday evening, February 21. Program: Erwin F. Smith, director of the National Bureau of Pathology addressed the Academy on his investigations into cancer, with stereopticon demonstration.

---

THE Medical Society of the County of Erie held its regular meeting in the rooms of the Society of Natural Sciences, Monday, February 20, 1911, under the presidency of D. V. McClure. After the business session Edward Clark discoursed on A plea for early operative treatment of rectal diseases.

The program read as follows: Some indications for the use of blood serum and for the use of blood transfusion, F. C. Busch; Double inguinal strangulated hernia—Krypt orchid—operation, L. G. Hanley; Report of cases of heart-block and Stokes-Adams syndrome, John Gray; Differential diagnosis of spinal diseases, by F. S. Crego.

Discussions followed the reading of papers.

---

THE Medical Society of the County of Erie at its Eighty-ninth annual meeting held Monday evening, December 19, 1910, elected the following named officers: president, D. V. McClure; first vice-president, Thomas H. McKee; second vice-president, Frank A. Helwig; secretary, Franklin C. Gram; treasurer, Albert T Lytle. A full account of the proceedings of the Society was published in the February number of the JOURNAL.

